# Interstitial lung diseases in the hospitalized patient

**DOI:** 10.1186/s12916-015-0487-0

**Published:** 2015-09-25

**Authors:** Supparerk Disayabutr, Carolyn S. Calfee, Harold R. Collard, Paul J. Wolters

**Affiliations:** Division of Pulmonary, Critical Care, Allergy and Sleep Medicine, Department of Medicine, University of California, Box 0111, San Francisco, CA 94143-0111 USA

**Keywords:** Acute exacerbation of IPF, Pulmonary fibrosis, Interstitial pneumonitis, Interstitial lung disease, Diffuse alveolar damage

## Abstract

**Background:**

Interstitial lung diseases (ILDs) are disorders of the lung parenchyma. The pathogenesis, clinical manifestations*,* and prognosis of ILDs vary depending on the underlying disease. The onset of most ILDs is insidious, but they may also present subacutely or require hospitalization for management. ILDs that may present subacutely include acute interstitial pneumonia, connective tissue disease-associated ILDs, cryptogenic organizing pneumonia, acute eosinophilic pneumonia, drug-induced ILDs, and acute exacerbation of idiopathic pulmonary fibrosis. Prognosis and response to therapy depend on the type of underlying ILD being managed.

**Discussion:**

This opinion piece discusses approaches to differentiating ILDs in the hospitalized patient, emphasizing the role of bronchoscopy and surgical lung biopsy. We then consider pharmacologic treatments and the use of mechanical ventilation in hospitalized patients with ILD. Finally, lung transplantation and palliative care as treatment modalities are considered.

**Summary:**

The diagnosis of ILD in hospitalized patients requires input from multiple disciplines. The prognosis of ILDs presenting acutely vary depending on the underlying ILD. Patients with advanced ILD or acute exacerbation of idiopathic pulmonary fibrosis have poor outcomes. The mainstay treatment in these patients is supportive care, and mechanical ventilation should only be used in these patients as a bridge to lung transplantation.

## Background

Interstitial lung diseases (ILDs) are diseases that afflict the lung parenchyma. The pathogenesis, clinical manifestations*,* and prognosis of ILDs depend on the specific underlying disease. Some patients require hospitalization during the course of their illness, and clinicians may face unique challenges while managing the hospitalized patient with ILD. Relevant considerations that are discussed in this manuscript include the ILDs commonly encountered in the hospital, diagnostic approaches, and medical management.

Recently, a multidisciplinary panel of American Thoracic Society/European Respiratory Society (ATS/ERS) members published a revised classification of ILDs based on their clinical, radiologic, and histopathologic findings (Table 1) [1]. The incidence and clinical course of ILDs are variable, depending on the underlying disease. For example, the overall incidence of idiopathic pulmonary fibrosis (IPF) in the United States is estimated to be 6.8 to 16.3 per 100,000 person-years [2], and the incidence increases with advancing age [2, 3]. Median survival for IPF patients is estimated to be 2 to 5 years from the time of diagnosis [4, 5]. In contrast, the annual incidence of cryptogenic organizing pneumonia has been estimated to be 2.0 per 100,000 person-years and the median survival 8 years from diagnosis [6].

**Table 1:** Classification of interstitial lung diseases

Classification	Clinical-radiological-pathological diagnosis	Histopathologic pattern
ILDs of known cause	Environmental or drug related	Depends on underlying disease
	Connective tissue disease related	Depends on underlying disease
	Hypersensitivity pneumonitis	
Idiopathic interstitial pneumonias (IIPs)		
Major IIPs		
Chronic fibrosing IPs	IPF	UIP
	Idiopathic NSIP	NSIP
Smoking-related IPs	DIP	DIP
	RB-ILD	Respiratory bronchiolitis
Acute/subacute IPs	COP	Organizing pneumonia
	AIP	Diffuse alveolar damage
Rare IIPs	Idiopathic LIP	LIP
	Idiopathic PPFE	Elastotic fibrosis of pleura and subpleural parenchyma
Unclassifiable IIPs		
Granulomatous lung disorders	Sarcoidosis	Non-necrotizing granuloma
Other forms of ILD	LAM	Cysts and proliferation of LAM cells
	PLCH	Proliferation of Langerhans cells
	Eosinophilic pneumonia	Depends on disease onset

*AIP* acute interstitial pneumonia, *COP* cryptogenic organizing pneumonia, *DIP* desquamative interstitial pneumonia, *IP* interstitial pneumonia, *IPF* idiopathic pulmonary fibrosis, *LAM* lymphagioleiomyomatosis, *LIP* lymphocytic interstitial pneumonia, *NSIP* nonspecific interstitial pneumonia, *PLCH* pulmonary Langerhans cell histiocytosis, *PPFE* pleuroparenchymalfibroelastosis, *RB-ILD* respiratory bronchiolitis-associated interstitial lung disease, *UIP* usual interstitial pneumonia

The clinical presentation of many ILDs is insidious; however, they may also present acutely. In some instances patients need to be hospitalized during the first manifestation of what ultimately proves to be a chronic ILD. Examples of ILDs that usually require hospitalization include acute exacerbations of idiopathic pulmonary fibrosis (AE-IPF), acute interstitial pneumonia (AIP), cryptogenic organizing pneumonia (COP), acute eosinophilic pneumonia (AEP), rapidly progressive or acute exacerbation of connective tissue disease-associated ILDs (CTD-ILD), and drug-induced ILDs.

### ILDs commonly requiring hospitalization: clinical presentation and prognosis

#### IPF and acute exacerbations of IPF (AE-IPF)

Idiopathic pulmonary fibrosis (IPF) is a fibrosing interstitial lung disease associated with aging [7]. Symptomatically, patients report progressive dyspnea and a nonproductive cough. Radiographically, high resolution computed tomography (HRCT) of the chest in a patient with IPF reveals bilateral subpleural reticulation, traction bronchiectasis, a paucity of ground glass opacities, and subpleural honeycombing [8]. The lung pathology of patients with IPF shows temporal heterogeneity of fibrosis that is accentuated subpleurally, with relatively normal lung centrally, microscopic honeycombing, and fibroblast foci. Median survival for IPF is just 3 years; progressive breathlessness and respiratory failure are the most common natural history [5].

Patients may be hospitalized at the time IPF is first recognized, often when the patient has an intercurrent infection, or may suffer an acute exacerbation of their disease (AE-IPF). AE-IPF is the most common cause of acute respiratory deterioration and death in IPF patients [9, 10]. In some instances AE-IPF may be due to a secondary cause, such as air pollution [11], microaspiration [12], or an intercurrent infection in up to one-third of patients [13]. AE-IPF can occur at any time during the course of disease. Although it may be the first manifestation of IPF [9, 10], the frequency of AE-IPF appears to be more common late in the disease course. AE-IPF is characterized by new or worsening of respiratory symptoms, typically in less than 30 days, accompanied by new lung opacities on chest imaging [14]. The characteristic HRCT of the chest shows new ground glass opacities with or without consolidation overlying the typical radiographic changes of IPF [14]. The most common pathological finding is diffuse alveolar damage (DAD) superimposed on the typical pathologic findings of IPF. The outcome of AE-IPF is poor, with a median survival post-AE of approximately 4 months [10, 15].

#### Connective tissue disease-associated ILDs (CTD-ILD)

Interstitial lung disease has been described in all types of connective tissue disease. Rheumatoid arthritis (RA), polymyositis/dermatomyositis (PM/DM), and systemic sclerosis (SSc) are the most common CTDs that develop an ILD [16]. The majority of patients with CTD-ILD have insidious onset of respiratory symptoms, including shortness of breath, dyspnea on exertion, and cough. Depending on the CTD, nearly all radiographic and histopathologic subtypes of ILD, including usual interstitial pneumonia (UIP), nonspecific interstitial pneumonia (NSIP), DAD, organizing pneumonia (OP), bronchiolitis, cystic lung disease, and diffuse alveolar hemorrhage have been reported in patients with CTD. For example, NSIP is the most common radiologic and histopathologic pattern found in patients with SSc or PM/DM [17], a UIP pattern is most common in patients with RA [18], and NSIP with or without areas of OP is most commonly found in antisynthetase syndrome [19, 20].

Similar to IPF patients, patients with CTD-ILD may develop subacute respiratory symptoms that occur either as a rapidly progressive CTD-ILD at presentation or an acute exacerbation of preexisting CTD-ILD [21]. The incidence and prevalence of rapidly progressive CTD-ILD varies depending on the underlying CTD, but is most common in PM/DM, especially the amyopathic subtype [22]. RA-ILD is the most common preexisting CTD-ILD that has acute exacerbations. Risk factors for acute exacerbation of CTD-ILD include advanced age, surgical lung biopsy, diagnosis of RA-UIP, and methotrexate treatment [21, 23, 24]. Acute exacerbations of CTD-ILD may occur independent of a flare of extrathoracic manifestations of the CTD and/or while patients are on immunosuppressive treatment. Clinical manifestations mimic conditions that can coexist with underlying CTD including pulmonary infection and treatment-related complications. The radiographic pattern of acute exacerbations of CTD-ILD shows new ground glass opacities superimposed on fibrotic changes that were evident previously. Similarly, lung pathology shows changes of DAD superimposed on a background of lung fibrosis. The outcome of acute exacerbations of CTD-ILD is poor, with hospital mortality rates of 50–100 % [21, 24].

#### Cryptogenic organizing pneumonia (COP)

Organizing pneumonia may have a known cause or may be cryptogenic (COP) [1]. In both cases, the presentation is usually subacute with symptoms mimicking infection. However, 10–15 % of patients with COP have a rapidly progressive course [25]. Common symptoms are a nonproductive cough, dyspnea on exertion, fever, and malaise. Radiographically, the HRCT shows patchy areas of consolidation in association with airways. Perilobular abnormalities (curved-like bands of parenchymal consolidation with blurred borders along the structures that surround the secondary pulmonary lobule) [26] or reversed halo sign (region of consolidation with central clearing) are highly suggestive of OP [27]. The dominant pathological finding on lung biopsy is intra-alveolar granulation tissue without hyaline membranes [28]. When confronted with a case of organizing pneumonia, it is important to rule out secondary causes (for example, cancer, drugs, CTD, hypersensitivity pneumonitis) before considering the case cryptogenic. The majority of patients with COP have complete clinical and radiological resolution with corticosteroid treatment. Pathological predictors of unfavorable outcome are coexistent lung fibrosis, which suggests the organizing pneumonia is not present in isolation but rather a feature of a more dominant ILD pattern such as NSIP [29].

#### Acute interstitial pneumonia (AIP)

AIP is an ILD of unknown cause that presents subacutely and characteristically progresses to severe hypoxemic respiratory failure. Chest radiograph and HRCT findings reveal bilateral airspace opacities in a pattern similar to those seen in patients with acute respiratory distress syndrome (ARDS) [30]. When caring for a patient with suspected AIP, it is critical to first rule out underlying causes of ARDS, which would be treated differently and according to the underlying ARDS risk factor (for example, sepsis, aspiration, pneumonia) [31]. Histopathologically, AIP demonstrates a pattern of DAD that is indistinguishable from acute lung injury of known cause. The prognosis for AIP is poor and worse than that of ARDS, with a reported mortality of greater than 50 % [32, 33]. The reported progression to fibrosis in a subset of patients may represent acute exacerbations of a previously unrecognized interstitial lung disease rather than progression of AIP to fibrosis [34].

#### Acute eosinophilic pneumonia (AEP)

AEP is an eosinophilic lung disease that can lead to hypoxemic respiratory failure requiring mechanical ventilation. Recent reports suggest that AEP may develop as a hypersensitivity reaction to tobacco smoke or dust exposure [35–37]. Patients present with symptoms of fever, cough, and shortness of breath, and a complete blood count may show peripheral eosinophilia. Radiographically, the HRCT shows bilateral ground glass opacities that are predominantly located in the lung periphery. Cell differential on bronchoalveolar lavage fluid typically contains greater than 30 % eosinophils. Lung histopathology shows marked infiltration of eosinophils in the lung interstitium and alveolar spaces with patchy areas of organizing pneumonia.

#### Drug-induced interstitial lung disease

Drug-induced ILD comprises 2–3 % of all ILDs [38]. More than 350 drugs have been reported to cause lung disease [39, 40]. The diagnosis of drug-induced ILD may be challenging because clinical and radiographic findings are similar to those of many pulmonary diseases. Clues to drug-induced ILD are that the onset of symptoms may correlate to time of first use of the medication (although this relationship is variable and the latency period can be quite long) and a high index of suspicion in all new cases of ILD. Depending on the implicated drug, nearly all radiographic and histopathologic types of lung disease, including NSIP, DAD, OP, eosinophilic pneumonia, pulmonary edema, and diffuse alveolar hemorrhage have been reported. NSIP is the most common pathologic pattern of drug-induced ILD.

#### Comorbidities

IPF and other fibrotic ILDs may be associated with comorbid illnesses, including pulmonary embolism, pulmonary hypertension, pulmonary infection, pneumothorax, left-sided heart disease, coronary artery disease, lung cancer, emphysema, gastroesophageal reflux disease (GERD), or sleep disorders. Progression of these comorbidities may be the cause of hospitalization and significantly impact patient outcomes [15, 41, 42]. Thus, patients with worsening of respiratory symptoms require evaluation for progressive disease and consideration that these comorbidities may be contributing [43].

### Discussion: diagnosis and management of acute manifestations of ILD

#### How are ILDs clinically differentiated in the hospitalized patient?

Patients with ILD can be hospitalized at the time the ILD is first recognized, or during the progression of an established ILD. When presenting for the first time, clinical, radiographic, and laboratory tests are required to differentiate the cause of the ILD. Historical clues include the patient having symptoms consistent with a connective tissue disease (such as joint pain, swelling, rash, Raynaud’s phenomenon, or muscle weakness), use of a medication that causes ILD, or risk factors for organizing pneumonia (medications, cancer, radiation therapy, inhalational injury). Useful laboratory tests include serologies to rule out a connective tissue disease, for example, tests for antinuclear antibody (ANA) and subtypes, rheumatoid factor (RF), cyclic citrullinated peptide (CCP), and synthetase antibodies. These tests should be considered even in patients without systemic symptoms of a CTD, since ILD can be the first manifestation of a CTD.

Chest imaging should be performed in all patients hospitalized with an ILD. Chest radiography is useful as an initial assessment of the amount of lung involvement, but is insensitive to new opacities, especially those overlying preexisting abnormalities. HRCT is more sensitive to detect radiographic abnormalities and does not require iodinated contrast. The pattern and distribution of abnormality on HRCT can aid in refining the differential diagnosis of the ILD and in some cases establish the diagnosis of a specific ILD. In patients without a prior history of ILD, HRCT features that suggest presence of a preexisting ILD are architectural distortion such as traction bronchiectasis or honeycombing. These findings can be used to differentiate AE of a chronic ILD from AIP or ARDS, which typically do not have traction bronchiectasis or honeycombing [44].

The most common cause for hospitalization of patients with previously diagnosed ILD is AE of preexisting ILD [45]. Clinical manifestations include acute progressive dyspnea, cough, with or without fever, and bilateral inspiratory crackles. These exacerbations may be due to progression of the underlying ILD. However, it is important to consider secondary causes such as a superimposed lung infection, left-sided heart failure, diffuse alveolar hemorrhage, or adverse effects of treatment [46]. Pulmonary function tests typically cannot be performed on patients suffering an AE of ILD due to the severity of their illness. If performed, they typically demonstrate progressive reduction of forced vital capacity (FVC) and diffusing capacity for carbon monoxide (DLCO).

#### What is the role of bronchoscopy?

Bronchoscopy with bronchoalveolar lavage (BAL) or transbronchial biopsy may be useful in cases where clinical history and chest imaging cannot establish a diagnosis. Prior to bronchoscopy, the risk of BAL should be carefully considered, because BAL can lead to worsening of hypoxemia, especially in spontaneously breathing patients. BAL can be used in patients with acute ILD or AE-IPF to confirm lung infection by bacterial culture and PCR for viruses. However, a negative BAL analysis does not definitively exclude infection. Its sensitivity is no better than 70 % for many infections. BAL analysis can be particularly useful in immunosuppressed patients with CTD-ILD, because infections such as *Pneumocystis jirovecii* in patients with CTD-ILD (especially dermatomyositis patients who are at particularly high risk for *Pneumocystis jirovecii* infection) may have a similar radiographic appearance to an acute exacerbation of CTD-ILD.

BAL cell count and differential may be useful in the diagnostic workup of the hospitalized patient with ILD. It is particularly helpful in establishing the diagnosis of AEP, where eosinophil counts greater than 30 % in BAL fluid are consistent with the diagnosis of AEP [36]. BAL cell count and differential are less helpful in patients with AIP or AE-IPF. The BAL cell count and differential for these diseases are typically characterized by an elevated percentage of neutrophils, and slight lymphocytosis. These cellular differentials are similar to those found in patients with bacterial or viral infections, restricting use of the cell differential in differentiating AE-ILD from an infection in an ILD patient.

Transbronchial biopsy is of limited utility in the management of the hospitalized ILD patient due to the small size of the tissue sample obtained, unless organizing pneumonia is suspected. In these patients, transbronchial biopsy yields the pathologic pattern of organizing pneumonia in up to 64 % of patients [47].

#### What is the role of surgical lung biopsy?

If medically necessary, surgical lung biopsy (SLB) can be used to differentiate interstitial lung disease of unknown cause, even in mechanically ventilated patients [48], and may lead to a change in treatment in hospitalized patients undergoing the procedure [48, 49]. Lung biopsy may be especially useful in hospitalized patients presenting with ILD for the first time when clinical history, laboratory tests, and chest imaging do not establish the diagnosis. Lung biopsy may also be useful in patients with a clinical presentation consistent with AIP or idiopathic ARDS, though a careful evaluation of the risks and benefits of empiric therapy versus biopsy must be undertaken first [50]. In cases of acute worsening of a previously recognized ILD, histopathology typically shows a DAD pattern overlying a pattern of fibrotic lung disease. Because the histopathological pattern of AEs is reliably consistent with abnormalities on HRCT and because 30-day mortality of AE patients is high, a surgical biopsy in patients with an AE of previously recognized ILD is rarely performed [45].

#### What pharmacologic treatments should be administered?

There are no well-designed, randomized, double blind placebo controlled trials to guide the pharmacologic therapy of the hospitalized ILD patient. Due to the challenge in differentiating lung infection from an acute presentation of ILD or acute exacerbation of preexisting ILD, treatment with broad-spectrum antibiotics should be considered in all patients. Nevertheless, clinical experience suggests that patients with specific ILDs improve with corticosteroid treatment. ILDs that appear to be steroid responsive are COP, AEP, some cases of CTD-ILD (importantly, high doses of corticosteroids are not recommended in SSc-ILD), and drug-induced ILDs. Response rates correspond to the underlying pathology, with eosinophilic pneumonia and OP being the most steroid responsive. The majority of patients with COP respond to corticosteroid treatment and have resolution of symptoms and radiographic abnormalities within weeks to months of initiating therapy [1, 51]. A typical initial dose of corticosteroid is 1 mg/kg/day of prednisone, which is then tapered to 20 mg/day after several weeks. Relapses may occur when treatment is stopped or rapidly tapered [51]. One year of treatment is often recommended [28, 51]. Corticosteroids are also useful in the management of AEP. Patients with AEP can have dramatic clinical improvement within 24–48 h after initiating corticosteroid treatment [36, 52].

Whether corticosteroids or immunosuppressants benefit patients with AE-IPF or AE of ILD other than IPF is uncertain. Corticosteroids may be beneficial when chest imaging suggests organizing pneumonia is present. Because of reports that azathioprine is associated with worse outcomes in patients with IPF or other forms of DAD, the use of cytotoxic medications should not be used for management of these patients [53]. In contrast to AE-IPF, there is a biologic rationale for treating AE of CTD-ILD patients with immunosuppressants. The driving force behind these diseases is believed to be hyperactivity of the immune system. Therefore, immunosuppressants such as mycophenolate mofetil, cyclophosphamide, or azathioprine may be useful in these patients.

#### What is the role of noninvasive ventilation and mechanical ventilation?

Patients hospitalized with an interstitial lung disease may have gas exchange abnormalities that require advanced ventilator support. The therapeutic options are high flow nasal cannula oxygen, noninvasive ventilation, or mechanical ventilation. There are a few small retrospective studies evaluating the role of noninvasive mechanical ventilation (NIV) in ILD patients presenting with acute respiratory failure. These studies report that use of NIV prevented endotracheal intubation in the minority of patients [54–57]. Of survivors in the NIV group, the majority died within 90 days following hospital discharge [56]. Furthermore, the majority of IPF patients failing NIV that subsequently were managed with mechanical ventilation died in the ICU [56, 57]. From these studies, it appears that the outcome of ILD patients requiring NIV is poor, though data from randomized controlled trials is lacking. However, NIV may provide benefit in a small subset of patients by preventing the need for mechanical ventilation.

If a patient is mechanically ventilated, the ventilator strategies applied often are similar to those used to manage patients with ARDS, because the pathological abnormalities of the lungs and alterations in lung mechanics are similar in these conditions [58]. Mechanical ventilation has been shown to improve oxygenation in 25 % of patients but did not influence levels of PaCO_2_ [59]. Unlike ARDS patients, patients with chronic ILD may have little or no recruitable lung and are prone to barotrauma due to overdistension of alveoli [58]. A case series of 94 patients with ILD and acute respiratory failure reported that low tidal volume was not associated with improvement of outcome and use of high levels of positive end-expiratory pressure (PEEP) was independently associated with lower short-term and long-term survival [60]. When patients with advanced lung fibrosis are managed with mechanical ventilation, the majority die in the intensive care unit without being liberated from the ventilator [54, 59]. Overall, these studies suggest that mechanical ventilation does not improve outcome in patients with AE-IPF or advanced lung fibrosis. The goals of mechanical ventilation should be carefully considered prior to offering it to patients with advanced ILD or an AE-IPF, as the prognosis is poor once mechanical ventilation is required to manage their illness.

#### What is the role of lung transplantation?

Pharmacological treatment and supportive measures may be ineffective for treating the hospitalized patient with ILD. This is especially true for patients with an acute worsening of IPF or those with advanced lung fibrosis. In these circumstances, lung transplantation may be the only remaining treatment option to consider in managing their disease. IPF is the second most common indication for lung transplantation [61]. Transplantation should be considered early in the hospitalization for patients with AE-IPF in order to arrange transfer to a transplant center for further evaluation and management. Contraindications to transplantation at many centers include age greater than 70, elevated BMI (>30 at most centers), active cancer, lack of dependable social support, recent substance abuse, or multiple medical comorbidities (Table 2) [62]. Mechanical ventilation or extracorporeal membrane oxygenation (ECMO) may be used as a bridging therapy to lung transplantation in select patients with ILD and severe gas exchange abnormalities [63]. When required prior to transplantation, mechanical ventilation has been associated with greater mortality in the first 6 months after transplant but does not appear to impact long-term survival [64].

**Table 2:** Contraindications for lung transplantation

Absolute contraindications	• Active malignancy in the last 2 years except for cutaneous squamous and basal cell tumors
	• Untreatable dysfunction of another major organ
	• Incurable extrapulmonary infection
	• Significant chest wall or spine deformity
	• Inability to follow through with medical therapy
	• Lack of dependable social support
	• Substance addiction within the last 6 months
Relative contraindications	• Age older than 70 years (65 in some centers)
	• Critical or unstable clinical condition
	• Severely limited functional status with poor rehabilitation potential
	• Colonization with highly resistant or virulent bacteria, fungi
	• Severe obesity (a body mass index > 30 kg/m^2^)
	• Severe or symptomatic osteoporosis

### When should palliative care be emphasized as the main treatment goal?

Because a significant percentage of patients hospitalized with advanced lung fibrosis or an AE of ILD succumb to their disease, the prognosis, probable outcome on mechanical ventilation, and role of palliative care should be considered once the diagnosis is established in these patients. When patients are hospitalized with acute respiratory failure, the role of life support and the risks of mechanical ventilation should be discussed and, when appropriate, palliative care interventions pursued as the main goals of therapy.

## Summary

Diagnosis of acute exacerbation of chronic ILD or acute ILD should be considered if indicated by clinical or radiological or histopathologic findings and other mimic conditions are already excluded. The mainstay treatment of these conditions is supportive care. Corticosteroids or immunosuppressants are indicated in some acute ILDs, including COP and AEP. Mechanical ventilation should only be offered in select cases, most notably patients with reversible processes or those considered candidates for urgent lung transplantation.

## Abbreviations

AIP: acute interstitial pneumonia
BAL: bronchoalveolar lavage
COP: cryptogenic organizing pneumonia
CTD-ILD: connective tissue disease-associated interstitial lung disease
DAD: diffuse alveolar damage
DLCO: diffusing capacity for carbon monoxide
DM: dermatomyositis
FVC: forced vital capacity
GERD: gastroesophageal reflux disease
HRCT: high resolution computed tomography
IIP: idiopathic interstitial pneumonia
ILD: interstitial lung disease
IPF: idiopathic pulmonary fibrosis
NIV: noninvasive mechanical ventilation
NSIP: nonspecific interstitial pneumonia
PM: polymyositis
RA: rheumatoid arthritis
SLB: surgical lung biopsy
SSc: systemic sclerosis
UIP: usual interstitial pneumonia
